# Supplementary material for: Tuning the catalytic CO hydrogenation to straight- and long-chain aldehydes/alcohols and olefins/paraffins
Source: Nat Commun. 2016 Oct 6;7:13058. doi: 10.1038/ncomms13058 (PMC5059682; doi:10.1038/ncomms13058)
Supplement: Supplementary Information — Supplementary Figures 1-12, Supplementary Tables 1-2, Supplementary Methods and Supplementary References [file ncomms13058-s1.pdf]

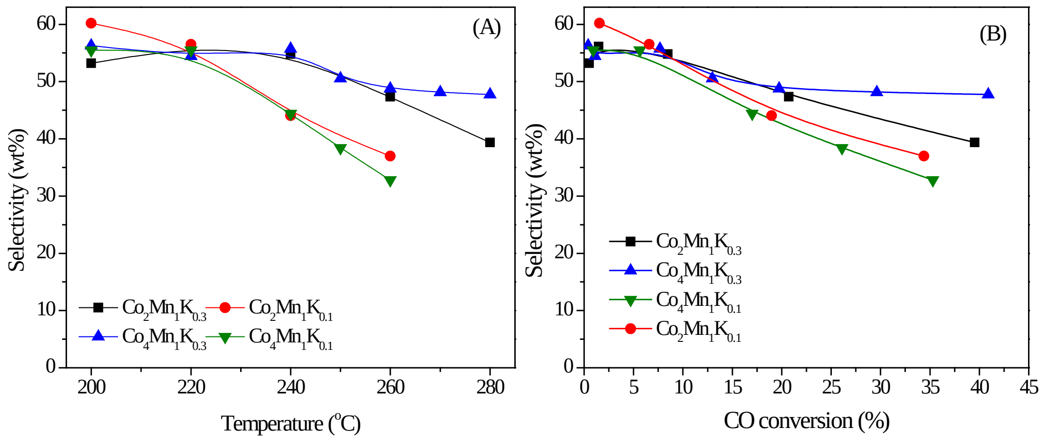

Supplementary Figure 1.

Total oxygenates selectivity (without considering CO_2 formation) as a function of reaction temperature (A) and CO conversion (B). A total pressure of 40 bar with a H_2/CO ratio of 1.5 was adjusted. The detailed reaction conditions are identical to those in Figure 1 of the main text.

The rational behind calculating total oxygenates selectivities without consideration of CO_2 formation is that in industrial applications frequently H_2-lean syngas (from coal or biomass) is being employed. Since most FT catalysts display water gas shift activities, the H_2/CO ratio (and therefore the CO_2 amounts) can be favorably adjusted. According to Figure S1, a total oxygenates selectivity up to ~50 wt% can be obtained at a CO conversion of more than 40%.

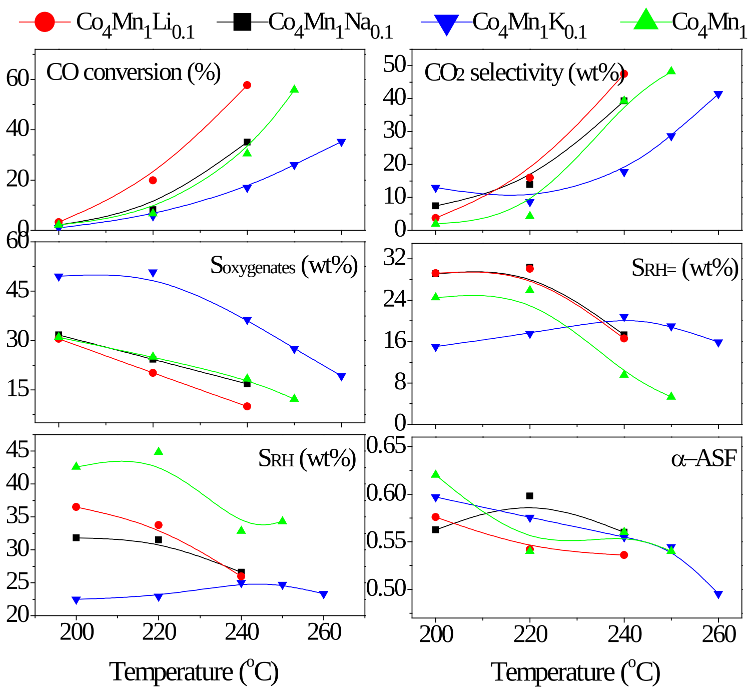

Supplementary Figure 2.

Effect of different alkali metals on the catalytic performance of Co_4Mn_1 catalysts in CO hydrogenation. Catalytic tests were carried out at p=40 bar, H_2/CO=1.5 and total gas flow=40 ml/min.

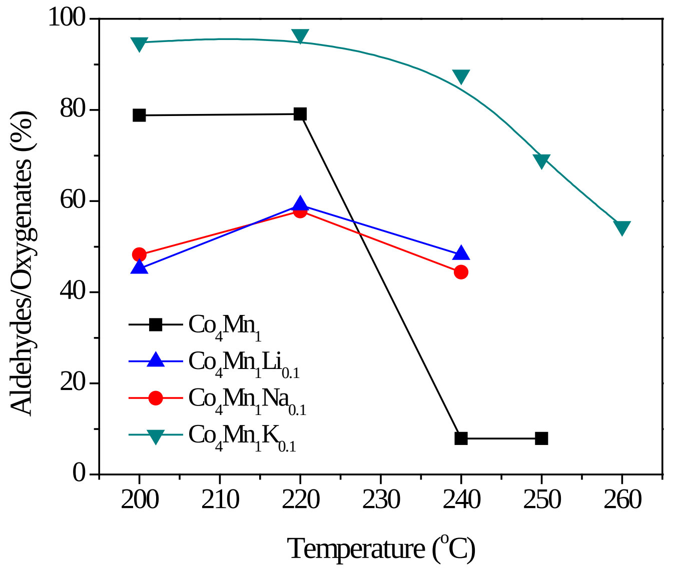

Supplementary Figure 3.

Selectivity of aldehydes in total oxygenates over different alkali containing Co_4Mn_1 catalysts.

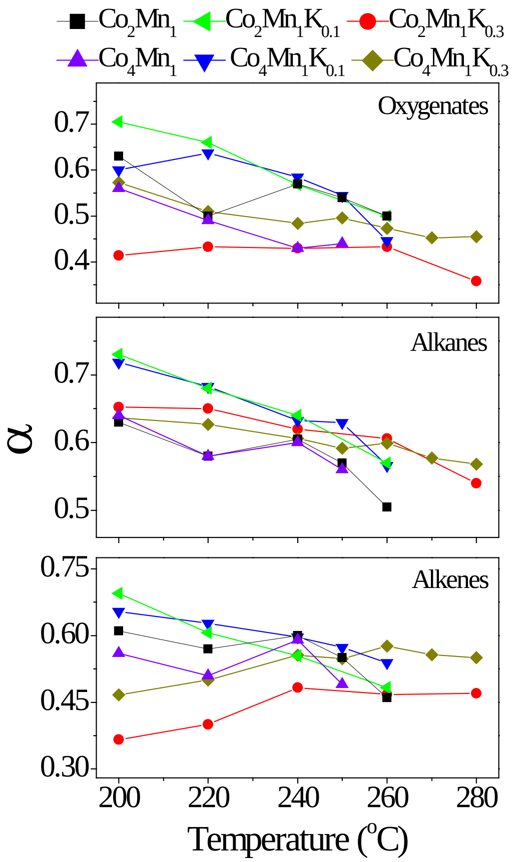

Supplementary Figure 4.

α-chain lengthening probabilities for oxygenates, alkanes and alkenes over various CoMnK catalysts. The α-values were calculated based on the Anderson-Schulz-Flory equation $W_n=n.(1-\alpha)^2.\alpha^{n-1}$ where W_n stands for the mass fraction of products containing n carbon atoms. The α values for oxygenates were calculated for C_{4+} because C_1-C_3 fractions do not follow the Schulz-Flory distribution. The α values for alkanes and alkenes were calculated for C_{2+} and C_{3+}, respectively.

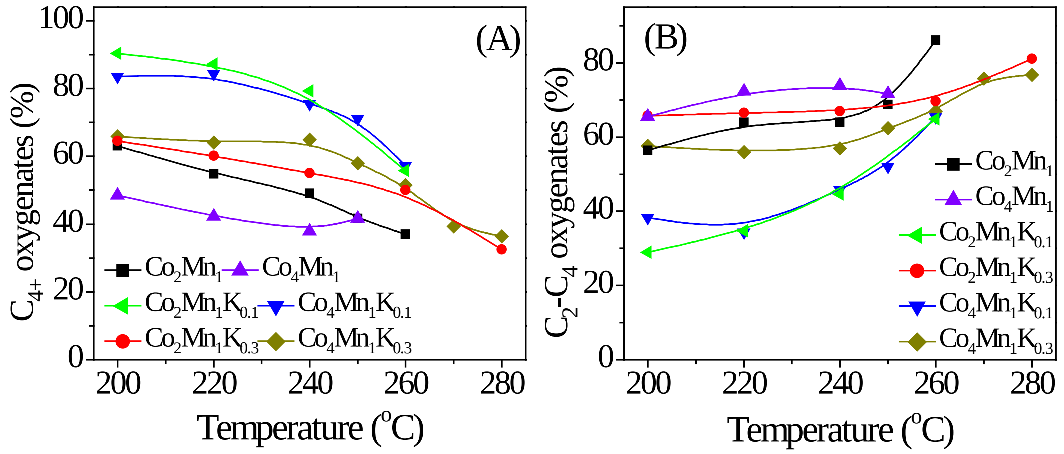

Supplementary Figure 5.

(A) selectivity of C_{4+} long chain oxygenates and (B) selectivity of C_2-C_4 short chain oxygenates over various catalysts and different temperature.

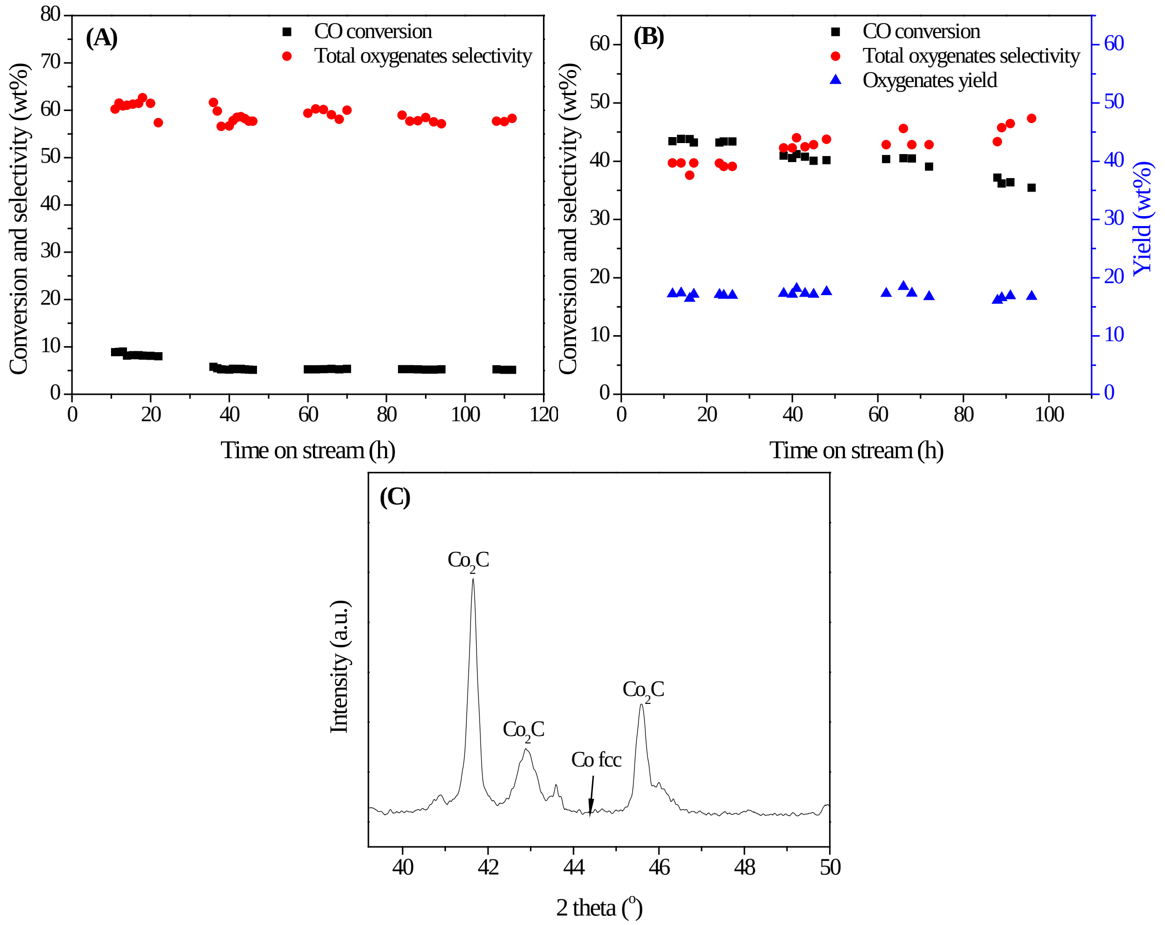

Supplementary Figure 6.

(A) and (B): Activity (CO conversion) and total oxygenates selectivity (without CO_2) as a function of time-on-stream for $Co_4Mn_1K_{0.1}$ catalyst at 220°C (Panel A) and 260°C (Panel B), 40 bar, H_2/CO=1.5.

It is seen that at both temperatures, the selectivity of total oxygenates (without CO_2) is remarkably stable within the investigated ranges of time-on-stream (between 56 to 62 wt% at 220°C and 40 to 47 wt% at 260°C, respectively). The selectivity of hydrocarbons (sum of paraffins and olefins) and CO_2 are also stable. At 220°C, the CO conversion during the measurements was always between 5 and 10%, but never below 5% within 112 h time-on-stream. At 260°C, the CO conversion slightly decreased from 44 to 35% after 96 h time-on-stream, however, the total oxygenates yield (CO conversion * oxygenates selectivity) remained strikingly stable (between 15 to 17 wt%).

(C): XRD pattern of the $Co_4Mn_1K_{0.1}$ catalyst after long-term stability testing for 96 h at 260°C. The XRD measurement was performed with the SiC-diluted catalyst as recovered from the reactor. The diffraction spectrum at 2 theta between 39-50° (typical diffraction region for Co metallic and Co carbides) shows clearly the occurrence of a Co_2C phase while the Co metallic phase as initially present in the virgin catalyst, is completely absent.

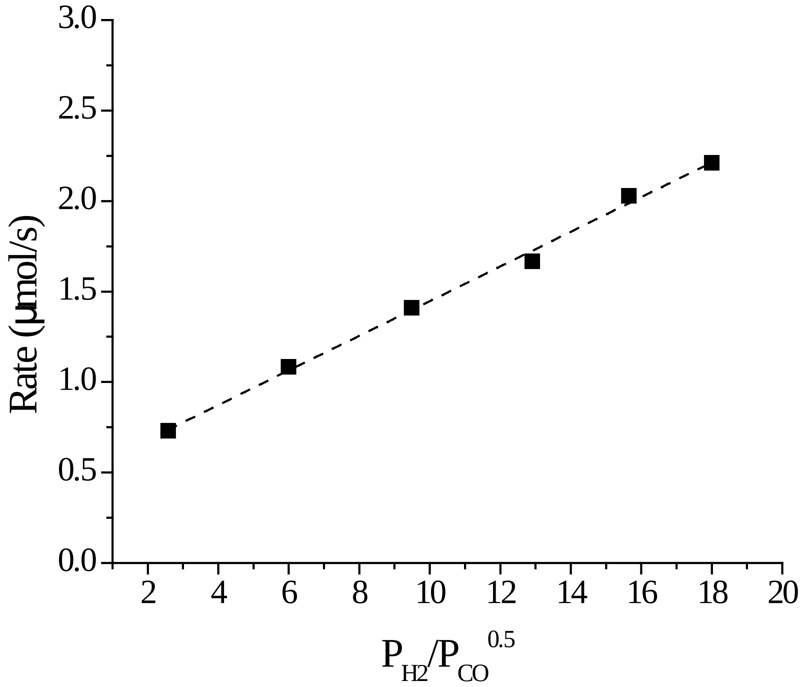

Supplementary Figure 7.

Kinetic analysis indicates that the rate of the reaction follows a first order dependence in the hydrogen pressure.

$$r = \mathrm{k}\, {P_{H2}}/{P_{CO}^{0.5}}$$

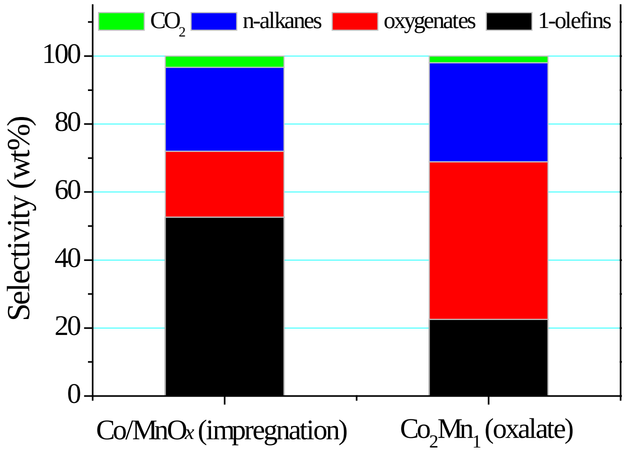

Supplementary Figure 8.

Comparison of selectivity between traditional supported Co/MnO_x catalyst (prepared by impregnation) with “unsupported” CoMn prepared through oxalate co-precipitation. To allow a comparison, the MnO_x support was prepared by thermal decomposition of Mn-oxalate. The Co particle size was around 10 nm for Co/MnO_x.

It is clearly seen that the selectivity of oxygenates is significantly higher for the Co_2Mn_1 (oxalate co-precipitated) catalyst than for Co nanoparticle (prepared from hot-injection of $Co_2(CO)_8$, see reference [2]) supported on MnOx (x = ~1.6, close to Mn_5O_8, prepared by thermal decomposition of Mn-oxalate).

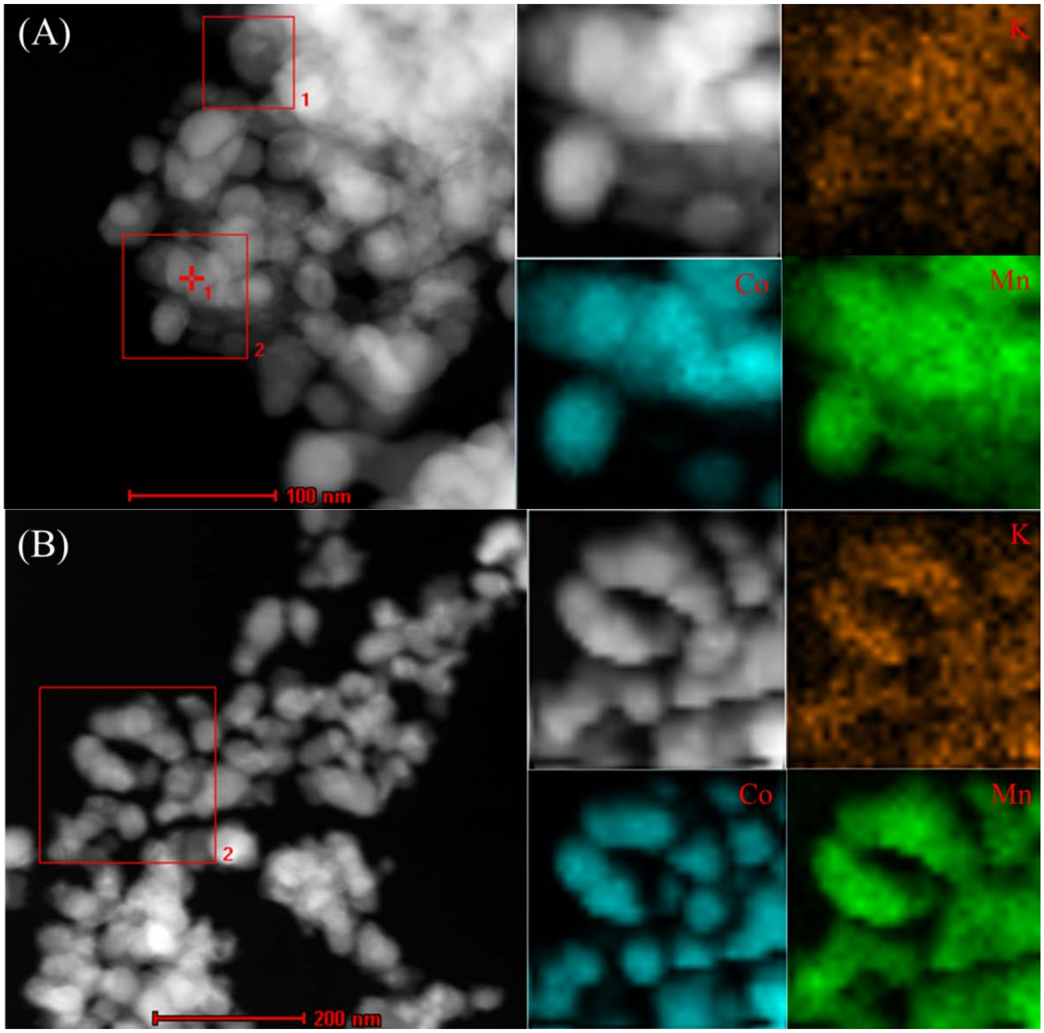

Supplementary Figure 9.

EDX chemical mapping of $Co_4Mn_1K_{0.1}$ catalyst (A) before and (B) after catalytic CO hydrogenation at 240 °C for 24 h.

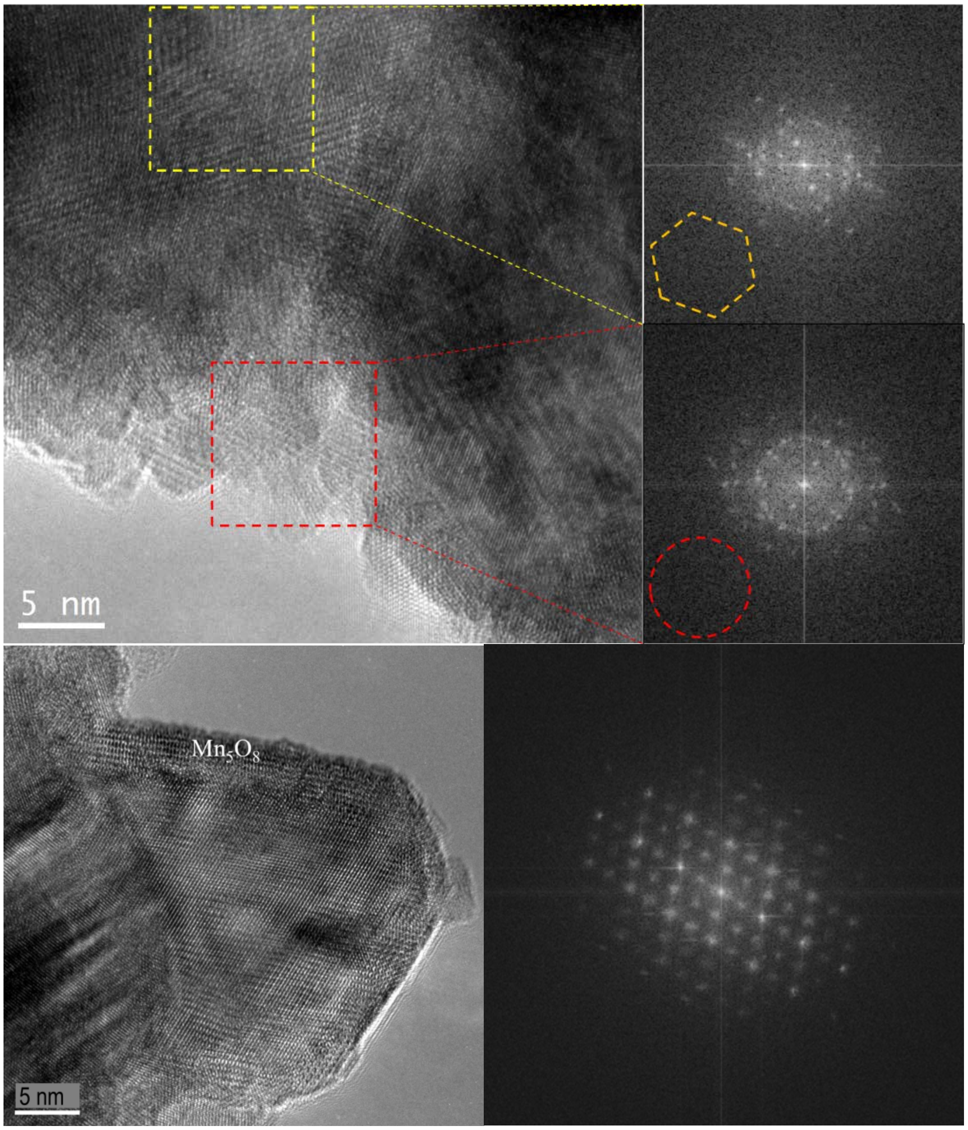

Supplementary Figure 10.

HRTEM images of the $Co_4Mn_1K_{0.1}$ catalyst before reaction.
Both fcc and hcp cobalt are identified (upper panel) through FFT of the selected regions.
In the lower panel, Mn_5O_8 is observed as a “shell” of a big cobalt (hcp) particle.

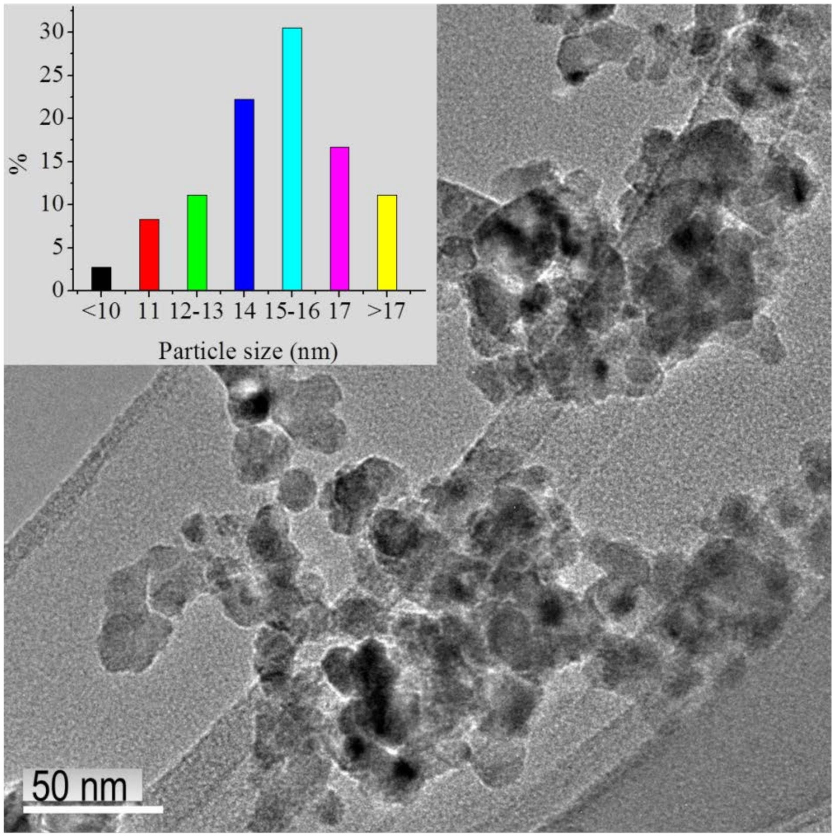

Supplementary Figure 11.

TEM image of $Co_4Mn_1K_{0.1}$ catalyst along with a particle size distribution histogram. More than 200 particles of the shown image as well as of other images were evaluated for their size so as to determine the percentage of each particle size range. These particles are composed of mainly cobalt with also certain amounts of manganese according to STEM-EDX (Figure S9).

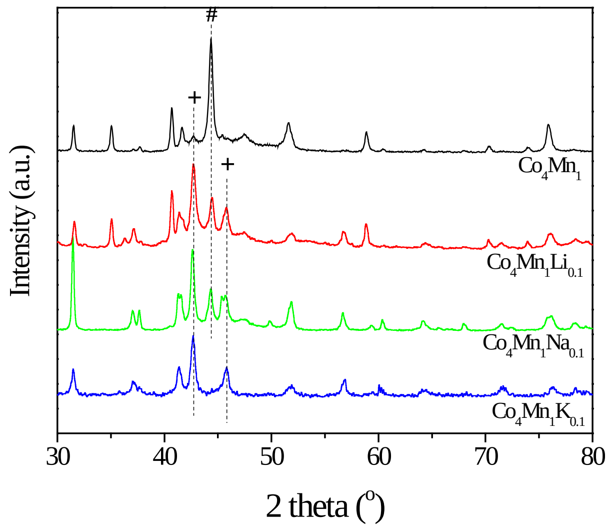

Supplementary Figure 12.

XRD patterns of Co_4Mn_1, $Co_4Mn_1Li_{0.1}$, $Co_4Mn_1Na_{0.1}$ and $Co_4Mn_1K_{0.1}$ catalysts after reaction for 24 h: T=240°C, p=40 bar, H_2/CO=1.5/1. (#): cubic Co and (+): Co_2C.

For Co_4Mn_1, fcc metallic Co was identified as being the major diffraction peak; peaks of Co_2C were not clearly attributable. The presence of alkali metals promoted the formation of Co_2C during reaction. With Li and Na, both metallic Co and Co_2C were observed, but for the K doped catalyst, metallic Co was completely absent. Other diffraction peaks in Figure S12 are associated with the Mn-oxide phase, alkali manganese mixed oxide etc. and will be discussed elsewhere.

Supplementary Table 1.

Catalytic performance of CoMn catalysts during CO hydrogenation.

Catalysts	CO conv. (%)	Selectivity (wt%)				RCHO in RO (%)
		RH	$R_=$	RO	CO_2	
Co_1Mn_2	4.5	36	34	24	6	75
Co_1Mn_1	4.9	37	32	25	6	76
Co_2Mn_1	6.5	39	30	25	6	78
Co_4Mn_1	6.6	45	26	25	4	79

Reaction conditions: 220°C, 40 bar, 4800 ml/g_{cat}/h, H_2/CO=1.5/1. RH: alkanes, $R_=$: alkenes, RO: oxygenates, RCHO: aldehydes.

Supplementary Table 2.

BET surface area of CoMnK catalysts with different relative amount of metal atom ratio.

Catalysts	Co_2Mn_1	$Co_2Mn_1K_{0.1}$	$Co_2Mn_1K_{0.3}$	Co_4Mn_1	$Co_4Mn_1K_{0.1}$	$Co_4Mn_1K_{0.3}$
S_{BET} (m^2/g)	74.5	47.0	37.6	69.2	46.1	33.2

Supplementary Methods

Catalyst preparation. Binary CoMn oxalates were co-precipitated by fast addition of a mixed solution of $Co(NO_3)_2 \cdot 6H_2O$ and $Mn(NO_3)_2 \cdot 4H_2O$ (100 ml) to an excess of $H_2C_2O_4 \cdot 2H_2O$ solution (150 ml) under vigorous stirring using acetone as solvent. Stirring usually continued for at least 5 min until the color of the precipitates appeared homogeneous. The atomic ratios of Co to Mn were adjusted to 1/2, 1/1, 2/1 and 4/1. Then the slurries were kept overnight for aging. After removal of the supernatant acetone the precipitate was centrifuged, dried overnight at 110°C and finally crushed and sieved so as to obtain a size fraction between 125 and 250 μm for characterization and high-pressure catalytic investigations.

The mixed-metal oxalate or hybrid precursors were treated thermally in the presence of hydrogen either directly in the high-pressure flow reactor (0.1 MPa H_2, 30 ml min^{-1} at 370 °C for 1 h) for catalytic testing, or in an atmospheric-pressure U-type flow reactor for characterization. Reduced catalysts were pyrophoric and had to be passivated (1% O_2 in Ar at 25 °C, for at least 30 min) before TEM and XRD characterization.

Catalyst characterization. X-ray diffraction (XRD) patterns of the catalysts were collected with a Cu Kα source using a Rigaku Miniflex-600 X-ray diffractometer operating at 40 mA and 35 kV in the continuous-scan mode with steps of 1 degree/min in a wide 2θ angle range from 20 to 80°.

(HR)TEM images and EDX chemical mapping of $Co_4Mn_1K_{0.1}$ before reaction were made at Zhejiang University of Technology using a Tecnai G2 F30 S-Twin TEM at an operating voltage of 300 kV. The HAADF STEM images for the $Co_4Mn_1K_{0.1}$ catalyst

after CO hydrogenation were collected at Pacific Northwest National Laboratory using a Titan 80-300™ scanning/transmission electron microscope S/TEM operating at 300 kV.

In-situ *Brunauer-Emmett-Teller* (BET) surface areas were measured using Ar as probe gas in a U-type flow reactor (made of quartz, ~5 cm^3 inner volume, equipped with a dead-end orifice for temperature measurement and a frit for sample deposition) connected to a quadrupole mass spectrometer (*Balzers-QMG 422*) via a capillary. The cross-section for non-specific Ar atom adsorption (σ_{Ar}=19.2 Å) was determined in calibration experiments with N_2 (σ_{N2}=16.2 Å) using Aerosil 200 (*Degussa*). Details of the methodological approach were described elsewhere[1].

Catalytic tests. For catalytic tests with varying H_2/CO pressure ratio, the H_2/CO flow rate was systematically changed while keeping the total pressure constant at p=40 bar. For each hydrogen incremental pressure increase, steady state conditions were first established before data were taken after 24 h time-on-stream.

The carbon balance has been double-checked by comparing the CO conversion (using an external standard) and the total carbon as detected in the products.

Supplementary References

1. Frennet, A., Chitry, V. & Kruse, N. In situ Measurement of the Surface Area During Catalyst Preparation: Development of a New Method. *Appl. Catal. A* **229**, 273-281 (2002).
2. Iablokov, V. *et al.* Size-Controlled Model Co Nanoparticle Catalysts for CO_2 Hydrogenation: Synthesis, Characterization, and Catalytic Reactions. *Nano Lett.* **12**, 3091-3096 (2012).
